# 45 Years of the mini-mental state examination (MMSE): A perspective from ibero-america

**DOI:** 10.1590/1980-5764-DN-2021-0097

**Published:** 2022-07-29

**Authors:** Miguel Gallegos, Melissa L. Morgan, Mauricio Cervigni, Pablo Martino, Jessie Murray, Manuel Calandra, Anastasia Razumovskiy, Tomás Caycho-Rodríguez, Walter Lizandro Arias Gallegos

**Affiliations:** 1 Universidad Católica del Maule, Talca, Chile.; 2 Pontifícia Universidade Católica de Minas Gerais, Belo Horizonte MG, Brazil.; 3 Consejo Nacional de Investigaciones Científicas y Técnicas, Buenos Aires, Argentina.; 4 Universidad Nacional de Rosario, Facultad de Psicología, Centro de Investigación en Neurociencias de Rosario, Santa Fe, Argentina.; 5 University of California Santa Barbara, California, USA.; 6 Arizona College of Nursing, Florida, USA.; 7 Universidad Privada del Norte, Lima, Perú.; 8 Universidad Católica San Pablo, Arequipa, Perú.

**Keywords:** Mental Status and Dementia Tests, History, Neuropsychology, Cognition, Latin America, Pruebas de Estado Mental y Demencia, Historia, Neuropsicología, Cognición, América Latina

## Abstract

The Mini-Mental State Examination (MMSE) was created by Marshal Folstein et al. in 1975 as an instrument for brief (5–10 min) assessment of mental status in hospitalized patients. It is considered the most widely used test for standardized cognitive assessment in the clinical setting, especially with the elderly population. It has countless translations in different languages, and according to the different international (PubMed) and regional (SciELO, Redalyc, and Dialnet) scientific databases, it has been widely used by the scientific community. This article describes the historical evolution of the MMSE, highlights its evaluative properties, and provides bibliometric data on its impact on scientific publications, with a special focus on Ibero-America.

## RESUMO

El Mini-mental State Examination (MMSE) fue creado por Marshal Folstein et al. en 1975 como un instrumento para la evaluación breve (5-10 minutos) del estado mental de pacientes hospitalizados. Se lo considera la prueba más utilizada para la evaluación cognitiva estandarizada en el ámbito clínico, especialmente con la población adulta mayor. Tiene innumerables traducciones a diferentes idiomas y de acuerdo con las diferentes bases de datos científicas internacionales (PudMed) y regionales (Scielo, Redalyc y Dialnet) se puede constatar que ha sido ampliamente utilizada por la comunidad científica. En este trabajo se describe la evolución histórica del MMSE, se destacan sus propiedades evaluativas y se indican datos bibliométricos acerca de su impacto en las publicaciones científicas, con especial énfasis en IberoAmérica.

## INTRODUCTION

It has just been 45 years since the publication of the Mini-Mental State Examination (MMSE): a brief assessment of cognitive performance^1^. The first construction of systematic, scientifically rigorous, psychological, and/or neuropsychological assessment instruments took place during the 20th century, even though there has always existed throughout human thought a need for the identification and description of psychological functions, such as character, temperament, personality, and intelligence^2^.

In this article, we give a brief history of the MMSE, detailing its advantages and disadvantages, and how it has impacted the neuroscientific community internationally, with a special emphasis on Ibero-America. Regional and international data are presented. The idea is to present a historical overview of the test, but not an in-depth review of all of its aspects, since there are several systematic reviews that could be consulted for this purpose — several of which are mentioned in this article.

### Function of the MMSE

This test was designed for brief application due to the excessive length of the existing tests in the mid-1970s^3^. It focused on strictly cognitive issues, leaving out questions related to psychiatric disorders or behavior. Despite its minor modifications over time, the test still consists of two parts. The first part evaluates questions related to orientation, memory, and attention, and the second part assesses verbal and written ability (requiring pencil and paper).

It is a test that has become one of the most widely used internationally for the diagnosis and clinical prognosis of cognitive impairment, mainly in elderly patients. An adaptation of the test given by telephone has even been created^4^. Recently, its performance as a tele-neuropsychological test was evaluated and indicated that there are no substantial differences when applied traditionally or remotely^5^.

Throughout its history, a series of advantages and disadvantages have been discovered. Its international acceptance and application, easy administration, short duration, application to large samples, and free access are among some of its advantages. Among the disadvantages are the multiplicity of versions, lack of exploration of all cognitive domains, lack of copyright for several years, and lack of sensitivity to cultural variations and the school level of the participants^6–10^.

The creators of the MMSE have recognized the importance of these criticisms and have attempted to improve the original version through increased precision and an indication of the need to comply with copyright; therefore, it is no longer available through public access and thus there is greater control over new translations and adaptations^11^.

### The international dissemination of the MMSE

Folstein’s work has been reported to be among the 50 most cited articles in the Web of Science database during the 20th century, receiving 15,000 citations as of January 2004^3^ and 19,721 citations up to February 2007^12^. A more recent study found 29,057 citations up to December 31, 2012^13^. It is also a test that has more than 70 translations into different languages^6^. As of August 18, 2021, in the international database PubMed, 20,032 related documents were retrieved for the keyword “MMSE.” Notably, 262 documents were retrieved with this keyword in the regional SciELO database. Table 1 shows the 10 journals with the highest number of mentions of MMSE.

**Table 1:** Journals containing articles on the MMSE.

N°	Name	Quantity
1	Dementia & Neuropsychologia	67
2	Arquivos de Neuro-Psiquiatria	42
3	Revista Brasileira de Geriatria e Gerontologia	12
4	Ciência & Saúde Coletiva	10
5	Brazilian Journal of Physical Therapy	8
6	Brazilian Journal of Psychiatry	8
7	Cadernos de Saúde Pública	8
8	Jornal Brasileiro de Psiquiatria	8
9	Acta Paulista de Enfermagem	5
10	Revista Médica de Chile	5

Source: SciELO (www.scielo.br), consultation August 18, 2021.

Two Brazilian journals have the highest concentration of these publications (109) according to the SciELO database, which has a predominance of Brazilian journals. However, a search of the Dialnet database, whose coverage is more Ibero-American, retrieved 298 articles, 109 theses, 3 book chapters, and 1 book related to the MMSE. Meanwhile, a search of the Redalyc database, with Latin American coverage, resulted in 376 articles: 221 in Spanish, 86 in English, and 69 in Portuguese. The disciplines referencing MMSE the most, according to Redalyc, are psychology with 146 papers, medicine with 138 papers, and health with 50 papers.

Different review papers on the MMSE have highlighted its wide use in cognitive assessment worldwide^3,9,14^. As noted, there have been several translations and adaptations of the instrument to various national contexts, and in several cases, different versions can be found, some validated and others not validated, as is the case with the Spanish-language versions for Latin America and Spain. This situation has made it difficult to compare the results of this instrument^5^. Comparisons can also be difficult due to cultural differences, for example, between applications in Spanish-speaking Latin America and Spanish-speaking communities in the United States^15,16^.

The first MMSE was created in Spain in the 1970s; since then, multiple validation efforts have been made^17^. However, despite Spanish being a common language to several countries, it is necessary to create regional versions, for example, in a country like Argentina, where there are normative references for different regions^15,18–21^.

In the Portuguese-language setting, and particularly in Brazil, the wide use and the existence of different versions of the MMSE have also been documented^22–25^, with 11 versions created for the study of elderly people, according to a review of September 2013^26^. However, according to this review, the most widely used version in Brazil was published by Bertolucci et al.^27^, in the *Arquivos de Neuro-Psiquiatria*, in 1994. Subsequently, recommendations were made for adaptation of the measure to hospitals, private practice, and community studies^23^. In Portugal, the first known translation and adaptation of the MMSE were also in 1994^28^, and since then, several adaptations have been created for various populations^29,30^.

This multiplicity of versions that have been generated over time at the international level has been criticized by the authors who originated the test, and they themselves have tried to rectify this problem by providing a guidance manual and a list of authorized versions and translations^31^. The proliferation of versions reflects not only the internationalization of the MMSE but also the need for a more precise instrument in the cognitive domain which is more in line with sociocultural variations.

The different versions that were established over time (e.g., 3MS, 3MS-R, SMMSE, MMSE-12, MMSE-20, and MMSE-37)^32–35^, many of them motivated by improving the assessment of cognitive abilities and covering aspects not covered in the initial version, did not achieve the popularity of the original test. This suggests at least two issues:

- The new versions probably did not achieve qualitatively different contributions and
- There is a strong weight of tradition inherited from the original version.

The MMSE has also served as a model and an inspiration for the development of other tests more specific to the assessment of cognitive abilities. Some have been presented as complementary and others are considered as alternatives, for example the following: Montreal Cognitive Assessment (MoCA), Addenbrooke;s Cognitive Examination (ACE), and Mini-Cog. In general, the MMSE is often used comparatively to assess the metric properties and diagnostic value of these new tests. In fact, several comparative studies have analyzed the advantages and disadvantages of each of the different cognitive tests and suggested the best test according to the cognitive function under assessment^36–38^. Overall, however, beyond the discrepancy in results, the MMSE remains a widely recommended and utilized instrument, although the MoCA test has become a substantial competitor to the MMSE, given its increasing use in research undertaken in Latin America^39,40^.

The MMSE has become a normative test at the international level, accepted by the neuroscientific community, and recommended by the main clinical practice guidelines on the assessment of cognitive impairment, particularly in older adults. Although it is widely used to test for Alzheimer’s or other types of dementia, it should be noted that it was not designed for that purpose. Although somewhat obvious, it should also be noted that the MMSE should never be viewed as a single assessment test, but rather as a tool in the overall clinical evaluation.

Despite certain limitations that have been noted regarding the multiplicity of versions and the comparability of results for different samples, the MMSE’s efficacy as a brief test remains valid for clinical practice. In addition, its application has been extended to population studies, since it can be rapidly administered and can be administered by non-specialized personnel.

One of the most notable aspects of the historical evolution of the MMSE lies not only in its frequent and widespread use as a cognitive assessment tool worldwide (including extensively in Latin American countries) but also in the fact that it has inspired the creation of new cognitive tests, many of which have been developed as complementary or alternative tests to the MMSE.
